# Design, Synthesis and Study of Nitrogen Monoxide Donors as Potent Hypolipidaemic and Anti-Inflammatory Agents

**DOI:** 10.3390/molecules25010019

**Published:** 2019-12-19

**Authors:** Panagiotis Theodosis-Nobelos, Georgios Papagiouvanis, Maria Pantelidou, Panos N. Kourounakis, Chrysoula Athanasekou, Eleni A. Rekka

**Affiliations:** 1 Department of Pharmacy, School of Health Sciences, Frederick University, Nicosia 1036, Cyprus; gpapagiou@gmail.com (G.P.); hsc.pm@frederick.ac.cy (M.P.); 2 Department of Pharmaceutical Chemistry, School of Pharmacy, Aristotelian University of Thessaloniki, 54124 Thessaloniki, Greece; panoskur@pharm.auth.gr; 3 Institute of Nanoscience & Nanotechnology, NCSR “Demokritos”, 15310 Agia Paraskevi Attikis, Athens, Greece; c.athanasekou@inn.demokritos.gr

**Keywords:** NO donors, synthesis, antioxidants, hyperlipidaemia, anti-inflammatory agents, lipoxygenase inhibition

## Abstract

Inflammation and oxidative stress are involved in cardiovascular diseases. Nitrogen monoxide participates in the regulation of endothelial processes. Thus, derivatives of classic nonsteroidal anti-inflammatory drugs (NSAIDs), trolox or cinnamic acids esterified with 2-(nitrooxy)ethanol were designed and studied. It was found that the nitrogen monoxide (NO) releasing activity was comparable to that of *S*-nitroso-*N*-acetylpenicillamine. The nitrooxy derivatives decreased potently lipid indices in the plasma of hyperlipidaemic rats (30–85%). All compounds presented increased anti-inflammatory activity in vivo, inhibiting carrageenan-induced rat paw oedema as high as 76%, up to six times higher than that of the parent acids. Lipoxygenase inhibitory activity was significant for most of them, although the parent molecules exerted a minor effect (IC_50_ > 0.2 mM). Those compounds incorporating an antioxidant structure inhibited rat microsomal membrane lipid peroxidation strongly and possessed radical scavenging activity. These results indicated that the described compounds could act at different targets in multifactorial diseases, further limiting the possible adverse effects of drug combinations.

## 1. Introduction

Oxygen-derived free radicals and inflammation are associated with a number of pathological conditions, including Alzheimer’s, Parkinson’s, cardiovascular, metabolic, gastrointestinal and autoimmune diseases [1].

Increased production of reactive oxygen species (ROS) can promote vascular dysfunction and atherogenesis. In patients with hyperlipidaemia and coronary artery disease, activation of NADPH oxidase and xanthine oxidase generates superoxide anion radical, contributing to oxidative stress. Oxidised low density lipoprotein cholesterol (LDL) is atherogenic. Hypercholesterolemia upregulates angiotensin II type 1 (AT1) receptors, further enhancing oxidative stress and vasoconstriction [2].

Classical non-steroidal anti-inflammatory drugs (NSAIDs) are widely used, although the produced non selective cyclooxygenase (COX) -1 and -2 inhibition is associated with gastric and renal complications [3]. Lipoxygenase (LOX) inhibitors can exert anti-inflammatory activity and limit the undesirable effects of COX inhibition [3]. The role of lipoxygenases on the generation of atherosclerotic plaques [4,5] and their implication in the inflammation of lipid tissue [6] is under investigation. Therefore, the interrelation of inflammation, hyperlipidaemia and ROS production has become evident.

Nitrogen monoxide (NO) participates in neuronal plasticity, memory and learning processes, vascular tone, inhibition of platelet aggregation and neutrophil chemotaxis. In cases of inflammatory and oxidative conditions, like diabetes, hypertension, hypercholesterolemia and atherosclerosis, uncoupling of nitrogen oxide synthase (NOS), propagation of oxidative stress, neutrophil and macrophage infiltration are observed [7]. The latter phenomena could be reversed by the administration of hypolipidaemic agents that could limit endothelial NOS uncoupling [8]. Furthermore, the reduction of the gastrointestinal toxicity of NSAIDs with the incorporation of NO releasing moieties has been reported [3,9].

**Figure 1:**
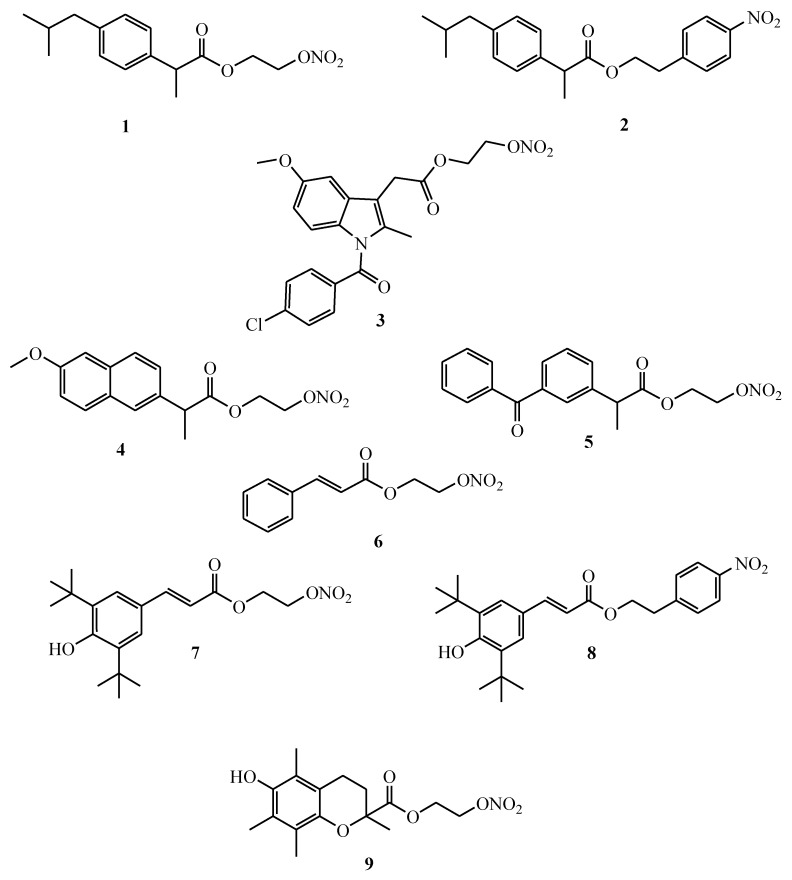
Structures of the synthesised compounds.

In view of the aforementioned evidence, adequate antioxidant and/or anti-inflammatory activity, combined with NO releasing potential could be considered useful in the treatment of complex diseases, such as those affecting the cardiovascular system. Therefore, we designed and synthesised a series of 2-(nitrooxy)ethanol esters with classic NSAIDs (compounds **1**, **3**–**5**), the well-known antioxidant trolox ((±)-6-hydroxy-2,5,7,8-tetramethylchromane-2-carboxylic acid), compound **9** and cinnamic acid ((*E*)-3-phenylacrylic acid) compound **6**, or BHCA ((*E*)-3-(3,5-di-*tert*-butyl-4-hydroxyphenyl)acrylic acid), compounds **7**, a cinnamic acid derivative possessing the molecular characteristics of both butylated hydroxytoluene (BHT) and cinnamic acid (Figure 1). 2-(Nitooxy)ethanol was selected, since we found that a chain of two carbon atoms between the carboxylate and the nitric ester groups favoured activity [9]. Two more compounds, one representing the NSAIDs group (**2**) and one from the antioxidant acids set (**8**), with a *p*-nitrophenyl moiety, were synthesised for comparison, due to their comparable lipophilic and electron withdrawing character.

Compound **1** is reported in the literature [10] as an agent promoting wound healing, without synthetic details or structure characterisation. Compound **5** has been synthesised previously [11] via a different method (nitration of 2-bromoethanol with a 70% HNO_3_-95% H_2_SO_4_ mixture gave nitrooxyethyl bromide, which yielded the final product after condensation with ketoprofen in the presence of Cs_2_CO_3_) and was tested for anti-proliferative activity using prostate cancer cells. All other compounds are novel. Compounds **1**, **2**, **5** and **9** are racemic.

The effect of the synthesised compounds on lipidaemic indices in the plasma of hyperlipidaemic rats was examined and the NO releasing ability was determined. The antioxidant activity, expressed as the inhibition of rat hepatic microsomal membrane lipid peroxidation, as well as the interaction with the stable radical 2,2-diphenyl-1-picrylhydrazyl (DPPH) was evaluated for selected compounds. The effects on carrageenan-induced rat paw oedema and the in vitro activity of soybean lipoxygenase were examined.

## 2. Results and Discussion

### 2.1. Synthesis

**Scheme 1:**
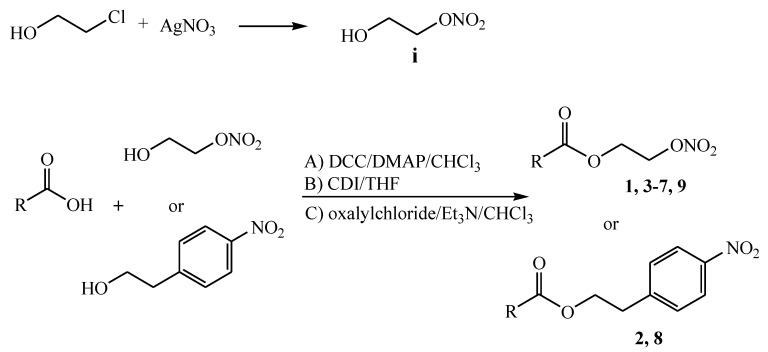
Synthesis of compounds **1**–**9**. A) Compounds **1**, **3**, **4**, **5**, **7**, **8:** Acid (1 mmol), 2-nitroxy-ethanol (2 mmol) or 2-(4-nitrophenyl)ethanol (2 mmol, compound **8**), DCC (1.5 mmol), DMAP (0.1 mmol), in CHCl_3_, 4 h, r.t. B) Compound **9**: Trolox (1 mmol), CDI, (1.1 mmol), tetrahydrofuran, stirring, 4 h. 2-Nitrooxy-ethanol (2 mmol), 24 h, r.t. C) Compounds **2**, **6**. Acid (1 mmol), oxalylchloride (3 mmol), 0–4 °C, CHCl_3_, stirring, 3 h. 2-Nitrooxy-ethanol (2 mmol), triethylamine (1.1 mmol), 3 h, r.t.

As shown in Scheme 1, the starting material **i** was prepared using silver nitrate and 2-chloroethanol [9]. Compound **i** or 2-(4-nitrophenyl)ethanol was esterified with (±) ibuprofen (compounds **1**, **2**), indomethacin (compound **3**), (+) naproxen (compound **4**), (±) ketoprofen (compound **5**), cinnamic acid (compound **6**), BHCA (compounds **7**, **8**) trolox (compound **9**) using three methods, in good yields (up to 80%). An excess of the corresponding alcohol was used due to the electron withdrawing effect of the nitrooxy group. The application of method **A** for the synthesis of the trolox derivative **9** failed to give the expected product, possibly due to steric reasons. Compound **9** was obtained (yield 74%) using CDI (method **B**), since this reaction is less sterically hindered.

### 2.2. Hypolipidaemic Effect

The pleiotropic effects of statins, used as hypolipidaemic drugs, include antioxidant ability and direct increase of eNOS activity, in addition to inhibition of 3-hydroxy-3-methylglutaryl coenzyme A (HMG-CoA) reductase [12]. Considering the aforementioned, compounds **1**, **3**–**7** and **9** were tested for anti-dyslipidaemic activity in Triton-induced hyperlipidaemia in rats. The systemic administration of Triton-WR1339 (tyloxapol) to rats induces a biphasic elevation of plasma cholesterol, atherogenic VLDL cholesterol and LDL cholesterol, as well as triglyceride levels. In phase I, plasma lipid levels increased sharply, after 24 h. Agents shown to be active in phase I are considered to interfere with cholesterol biosynthesis [13,14]. Results are shown in Table 1.

All tested compounds strongly decreased lipidaemic indices, especially cholesterol levels (LDL and total, 61–85% and 55.4–78.2%, respectively). The animals appeared normal, macroscopically and by autopsy, 24 h post administration. The activity of compounds was higher than that of the relevant parent acids at a much lower dose. In this experiment, it was confirmed that, although simvastatin profoundly lowered cholesterol, being comparable to the synthesised compounds, it had no effect on plasma triglyceride levels. Indeed, it has been reported that statins mainly lower serum LDL levels, whereas a serum triglyceride decrease is a minor effect [15]. The synthesised compounds significantly decreased triglyceride plasma concentrations as well (30–54.4%).

### 2.3. Nitrogen Monoxide Release

The ability of compounds **1**, **3**–**7** and **9** to release NO in vitro, at different concentrations is shown in Table 2. Compounds **2** and **8** were not included, because they were not possible NO donors. A linear increase in the amount of released NO was observed with increasing compound concentration.

With the small deviation of compounds **4** and **7**, compounds showed similar NO donating ability reaching up to 24 μΜ ΝΟ/100 μΜ compound. NO release from *S*-nitroso-*N*-acetylpenicillamine (SNAP), a known potent NO donor, was found to be 56.3μΜ / 100 μΜ.

Compounds with NO-releasing activity could be useful especially in cardiovascular complications. It is known that the NO precursor L-arginine reduced atherosclerosis in LDL receptor knockout hypercholesterolaemic mice, and this activity was inhibited by NOS inhibitors [16].

### 2.4. Lipoxygenase Inhibitory Activity

Lipoxygenases form the second major path of arachidonic acid metabolism. In atherogenic plaques considerable lipoxygenase activity has been observed [6], implicated in adipose tissue inflammation and atherosclerosis [17].

Although there are several lipoxygenase enzymes, it is believed that all enzymes share a common mechanism, i.e., the stereo- and region- specific peroxidation of arachidonic or linoleic acid by molecular oxygen [18]. Soybean lipoxygenase 1-B can use arachidonic acid as a substrate, with about 15% of the activity using linoleic acid.

The ability of compounds to inhibit lipoxygenase, presented as IC_50_ values towards soybean lipoxygenase 1-B, using linoleic acid as a substrate, after 7 min of incubation, is demonstrated in Table 3. The IC_50_ of nordihydroguaiaretic acid (NDGA), an antioxidant compound acting as a nonspecific inhibitor of lipoxygenase, was also included as a reference together with ibuprofen, ketoprofen and trolox. When linoleic acid was used at 1 mM, a concentration higher than the saturating substrate concentration, no inhibition was observed, under the same experimental conditions. These results indicate that the examined compounds act as competitive inhibitors of lipoxygenase, since inhibition is diminishing by increasing substrate concentration.

The majority of the compounds showed considerable activity, except for compound **4**. Trolox derivative **9** appeared active while trolox itself had an IC_50_ higher than 300 μΜ. Interestingly, compounds **2** and **8** were more active than **1** and **7**, respectively. At first sight, this might be attributed to the higher lipophilicity of the former compound (Table 3). However, the clog*P* value of **2** (5.92) was very close to that of compound **7** (6.16), but **2** had about double the activity of **7**. It has been reported that a 4-nitro group on a phenyl ring is among selective groups for 5-lipoxygenase inhibition [19]. Furthermore, di-*tert*-butylphenol derivatives, like tebufelone and darbufelone, are considered to comprise a class of dual COX-2/5-LOX inhibitors [19]. It was previously shown by us [20] that this structural moiety contributed to increased lipoxygenase inhibitory activity.

Among the nitric ester derivatives, it seems convincing that the more rigid molecules are less active. Thus, compound **4** was practically inactive, while compound **5**, although equally lipophilic with **4**, was quite active. Similarly, activity was lower for compounds **3** and **9**. Since we showed that these derivatives acted as competitive inhibitors of lipoxygenase, they were expected to occupy the hydrophobic cavity at the active site, which was intended for the flexible molecules of arachidonic or linoleic acid. Therefore, more flexible, lipophilc molecules are more active than the less flexible analogues, and the least lipophilc compound **6** had low activity.

### 2.5. Antioxidant and Radical Scavenging Activity

The antioxidant activity of compounds was estimated by the inhibition of rat hepatic microsomal membrane lipid peroxidation, induced by Fe^+2^/ascorbate, estimated as 2-thiobarbituric acid (TBA) reactive material, and by their interaction with the *N*-centred 1,1-diphenyl-2-picrylhydrazyl (DPPH) stable free radical [14]. Compounds **1**–**6** had minor antioxidant activity (inhibition of lipid peroxidation was less than 25% at 1 mM). The percent interaction with DPPH and the IC_50_ values of compounds **7**–**9** on rat hepatic microsomal membrane lipid peroxidation, after 45 min of incubation is shown in Table 4.

Compound **9** was almost an eleven-fold stronger inhibitor of lipid peroxidation than trolox and of similar activity with trolox as a DPPH radical scavenger. It is widely known (e.g., [20]) that the antioxidant activity of phenolic compounds is due to the phenolic hydrogen abstraction and can be enhanced by the extended conjugation of the chroman ring or the cinnamic structure, resulting in radical stabilisation. This effect may be further improved by the proper lipophilicity, which would permit an effective approach to the lipid phase.

**Figure 2:**
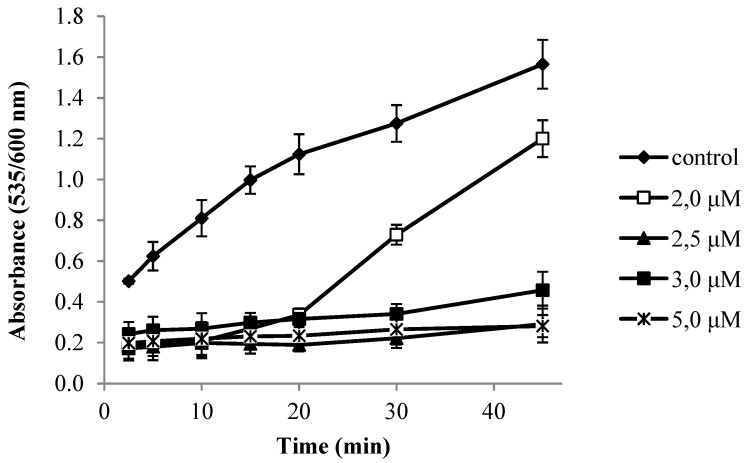
Effect of compound **9** (various concentrations) on the time course of lipid peroxidation.

The time course of lipid peroxidation, as affected by various concentrations of **9** is shown in Figure 2. A long lag period of about 20 min, characteristic of chain-breaking antioxidants, was observed.

Compound **7** showed considerable activity in both experiments. The lower protective effect of **8** against lipid peroxidation, compared to **7**, can be attributed to the lower solubility of **8** in the aqueous environment of the experiment. Compounds **7** and **8** had about the same reducing activity in the ethanolic medium of the DPPH test. Taken together, it could be suggested that these compounds may be able to offer protection against a free radical attack by direct inhibition of lipid peroxidation as well as by inhibiting lipoxygenase, considered to play a significant contributory role to atherosclerosis [21].

### 2.6. In Vivo Anti-Inflammatory Activity

The carrageenan-induced paw oedema is a well-known and widely used model of acute inflammation. The response to carrageenan inflammation may be divided into three phases. In the first phase (after 1.5 h), histamine and serotonin are released, the second phase (1.5–2.5 h after carrageenan administration) is mediated by kinins, whereas, in the third phase, more than two hours post administration, neutrophil infiltration, prostaglandin production and release of pro-inflammatory cytokines are involved [22]. In this work, oedema was measured 3.5 h post injection.

The effect of the synthesised compounds on acute inflammation, applying the carrageenan paw oedema model, as well as the anti-inflammatory activity of some classic NSAIDs used as a reference, is shown in Table 5.

The synthesised compounds demonstrated more than 50% oedema inhibition, except for compound **3**. This increase, compared with the parent NSAIDs was more than six fold higher for the naproxen derivative **4**, while **1** and **5** were about two times more active than ibuprofen and ketoprofen, respectively. It seems that esterification with 2-(nitrooxy)ethanol generally increased the anti-inflammatory effect of the NSAIDs. This molecular modification also added anti-inflammatory activity to the antioxidant acids and cinnamic acid. It has been previously reported by us that esters or amides of several NSAIDs, e.g., with 2-methoxy-4-methyl-phenol or thiomorpholine, enhanced the anti-inflammatory activity of the parent molecules [23,24] and that antioxidant acids such as trolox yield potent anti-inflammatory agents if they are esterified, e.g., with cinnamyl alcohol [20], while butylated hydroxytoluene (BHT) has been found devoid of any anti-inflammatory activity [9]. It has also been shown than the effect of a number of NSAID esters is not due to hydrolysis of the ester group [23].

## 3. Materials and Methods

### 3.1. General

All commercially available chemicals of the appropriate purity were purchased from Merck (Kenilworth, NJ, U.S.A.) or Sigma ((St. Louis, MO, U.S.A.). The IR spectra were recorded on a Perkin Elmer Spectrum BX FT-IR spectrometer (Waltham, MA, U.S.A.). The ^1^H NMR and ^13^C NMR spectra were recorded using a BRUKER Avance III-300 MHz (Billerica, MA, U.S.A.) or an AGILENT DD2-500 MHz ((Santa Clara, CA, U.S.A.) spectrometer. Chemical shifts were reported in *δ* (ppm) and signals were given as follows: s, singlet; d, doublet; t, triplet; m, multiplet. Melting points (mp) were determined with a MEL-TEMPII apparatus, Laboratory Devices, Sigma-Aldrich (Milwaukee WI, U.S.A) and were uncorrected. The microanalyses were performed on a Perkin-Elmer 2400 CHN elemental analyser (Waltham, MA, U.S.A.). Thin-layer chromatography (TLC silica gel 60 F_254_ aluminium sheets, Merck (Kenilworth, NJ, U.S.A.) was used to follow the reactions and the spots were visualised under UV light.

### 3.2. Synthesis

#### 3.2.1. 2-Nitrooxy-Ethanol [9]

Silver nitrate (35 mmol) was added to a solution of 2-chloroethanol (23 mmol) in acetonitrile (40 mL). The reaction mixture was stirred at room temperature overnight and was light protected. Then, the reaction mixture was filtered and the volatiles were removed under reduced pressure. The residue was dissolved in diethyl ether and washed with saturated NaCl solution. The organic layer was dried over calcium chloride, filtered, and concentrated.

Pale yellow oil, yield 16%. IR (film) *v*: 3400, 2900, 1630 cm^−1^_._^1^H NMR (CDCl_3_) *δ*: 2.00 (s, 1H, O**H**), 3.90–4.00 (t, *J* = 5 Hz, 2H, C**H**_2_OH), 4.50–4.60 (t, *J* = 5 Hz, 2H, C**H**_2_ONO_2_).

#### 3.2.2. General Procedures for the Synthesis of Compounds **1**–**9**

(A) Compounds **1**, **3**, **4**, **5**, **7**, **8:** The corresponding acid (1 mmol) and 2-nitrοoxy-ethanol (2 mmol) or 2-(4-nitrophenyl)ethanol (2 mmol, for compound **8**) were mixed in CHCl_3_. Then, *N,N’*-dicyclohexylcarbodiimide (DCC, 1.5 mmol) and *N,N*-dimethylaminopyridine (DMAP, 0.1 mmol) were added and dissolved in the same solvent. The mixture was stirred for 4 h at ambient temperature. The final compounds were isolated with flash column chromatography using mixtures of petroleum ether and ethyl acetate.

(B) Compound **9**: Trolox (1 mmol) and carbonyldiimidazole (CDI, 1,1 mmol) in tetrahydrofuran were mixed, stirred for 45 min and 2-nitrooxy-ethanol (2 mmol) was added and stirred for 24 h at ambient temperature. The solvent was distilled off, the residue was dissolved in ethyl acetate, washed with water, 5% aqueous HCl and NaHCO_3_ solutions and dried (Na_2_SO_4_). The final compound was isolated with flash column chromatography using mixtures of petroleum ether and ethyl acetate as eluents.

(C) Compounds **2** and **6**: The corresponding acid (1 mmol) was dissolved in CHCl_3_ and oxalylchloride (3 mmol) was added at 0–4 °C. After stirring for 3 h, the volatile compounds were distilled off. 2-nitrooxy-ethanol (2 mmol) and triethylamine (1.1 mmol), in the same solvent, were added at 0–4 °C and left for 3 h. The mixture was filtered, washed with water, 5% aqueous NaHCO_3_, saturated NaCl solutions and dried (Na_2_SO_4_). The final compounds were isolated with flash column chromatography with mixtures of petroleum ether and ethyl acetate as eluents.

*(±)-2-(Nitrooxy)ethyl 2-(4-isobutylphenyl)propanoate* (**1**): Flash column chromatography (petroleum ether/ethyl acetate, 40/1). Viscous transparent liquid, yield 85 %. IR (film) *ν*: 3052, 3024 (C-H aromatic), 2957, 2910, 2870 (C-H alkyl), 1739 (C=O ester), 1634 (N=O), 1513 (C-C aromatic) cm^−1^. ^1^H NMR (CDCl_3_), *δ*: 0.93 (d, 6H, *J* = 6.6 Hz, -CH-(CH_3_)_2_), 1.53 (d, 3H, *J* = 7.2 Hz, O=C-CH-C**H_3_**), 2.00–1.81 (m, 1H, -CH_2_C**H**-(CH_3_)_2_), 2.48 (d, 2H, *J* = 7.1 Hz, -C**H_2_**CH-(CH_3_)_2_), 3.75 (q, 1H, *J* = 7.1 Hz, O=C-C**H**-CH_3_), 4.45–4.29 (m, 2H, -O-CH_2_-C**H_2_**ONO_2_), 4.73–4.55 (m, 2H, -O-C**H_2_**-CH_2_ONO_2_), 7.12 (d, 2H, *J* = 8.0 Hz, phenyl C3, C5), 7.21 (d, 2H, *J* = 8.0 Hz, phenyl C2, C6). ^13^C NMR (CDCl_3_) *δ*: 18.32 (1C, O=C-CH-**C**H_3_), 22.35 (2C, -CH**_2_**CH-(**C**H_3_)_2_), 30.15 (1C, -CH**_2_C**H-(CH_3_)_2_), 44.85 (1C, O=C-**C**H-CH_3_), 45.00 (1C, -**C**H**_2_**CH-(CH_3_)_2_), 60.35 (1C, -O-**C**H_2_-CH_2_ONO_2_), 70.30 (1C, -O-CH_2_-**C**H_2_ONO_2_), 127.06 (2C, phenyl C2, C6), 129.39 (2C, phenyl C3, C5), 137.05 (1C, phenyl C4), 140.78 (1C, phenyl C1), 174.34 (1C, O-**C**=O). Anal. Calculated for C_15_H_21_NO_5_x0.3H_2_O: C, 59.91; H, 7.24; N, 4.66. Found: C, 59.73; H, 7.15; N, 4.86.

*(±)-4-Nitrophenethyl 2-(4-isobutylphenyl)propanoate* (**2**): Flash column chromatography (petroleum ether/ethyl acetate, 25/1 and gradually 10/1). Viscus liquid, yield 75%. IR (nujol) *ν*: 1723 (C=O ester), 1606, 1599 (C-C aromatic), 1623 (N=O) cm^−1^.^1^H NMR (CDCl3) *δ*: 0.94 (d, 6H, *J* = 6.5 Hz, -CH**-(CH_3_)_2_**), 1.48 (d, 3H, *J* = 7.1 Hz, O=C-CH-C**H_3_**), 1.89 (dt, 1H, *J* = 13.2, 6.5 Hz, -CH_2_C**H**-(CH_3_)_2_), 2.50 (d, 2H, *J* = 7.1 Hz, -C**H_2_**CH-(CH_3_)_2_), 2.99 (t, 2H, *J* = 6.2 Hz, -O-CH_2_-C**H_2_**-Ar), 3.67 (q, 1H, *J* = 7.1 Hz, O=C-CH-C**H_3_**), 4.50–4.23 (m, 2H, -O-C**H_2_**-CH_2_-Ar), 7.09 (d, 2H, *J* = 7.7 Hz, phenyl C3, C5), 7.14 (d, 2H, *J* = 7.9 Hz, phenyl C2, C6), 7.21 (d, 2H, *J* = 8.0 Hz, nitrophenyl C3, C5), 8.07 (d, 2H, *J* = 8.0 Hz, nitrophenyl C2, C6). ^13^C NMR (CDCl_3_) *δ*: 18.22 (1C, O=C-CH-**C**H_3_), 22.34 (2C, -CH**_2_**CH-(**C**H_3_)_2_), 30.18 (1C, -CH**_2_C**H-(CH_3_)_2_), 34.83 (1C, O-CH_2_-CH_2_-), 44.95 (1C, O=C-**C**H-CH_3_), 45.06 (1C, -**C**H**_2_**CH-(CH_3_)_2_), 63.93 (1C, O-CH_2_-CH_2_-), 123.53 (2C, nitrophenyl C3, C5), 127.12 (2C, phenyl C2, C6), 129.31 (2C, nitrophenyl C2, C6), 129.67 (2C, phenyl C3, C5), 137.39 (1C, phenyl C4), 140.73 (1C, phenyl C1), 145.68 (1C, nitrophenyl C4), 146.69 (1C, nitrophenyl C1), 174.41 (1C, O-**C**=O). Anal. Calculated for C_21_H_25_NO_4_x0.9H_2_O: C, 67.84; H, 7.27; N, 3.77. Found: C, 67.86; H, 7.46; N, 3.74.

*2-(Nitrooxy)ethyl 2-(1-(4-chlorobenzoyl)-5-methoxy-2-methyl-1H-indol-3-yl)acetate***(3)**: Flash column chromatography (petroleum ether/ethyl acetate, 10/1).Yellow powder, yield 50%, mp 83–84 °C. IR (nujol) *ν*: 1743 (C=O ester), 1684 (C=O amide), 1604, 1590 (C-C aromatic) cm^−1^. ^1^H NMR (CDCl_3_) *δ*: 2.41 (s, 3H, C**H_3_**-C-N-), 3.73 (s, 2H, -C**H_2_**-C=O), 3.87 (s, 3H, -O-C**H_3_**), 4.46–4.38 (m, 2H, -O-CH_2_-C**H_2_**ONO_2_), 4.75–4.60 (m, 2H, -O-C**H_2_**-CH_2_ONO_2_), 6.71 (dd, 1H, *J* = 9.0, 2.4 Hz, indole C6), 6.91 (d, 1H, *J* = 9.0 Hz, indole C7), 6.98 (d, 1H, *J* = 2.4 Hz, indole C4), 7.50 (d, 2H, *J* = 8.6 Hz, chloro-benzoyl C3, C5), 7.69 (d, 2H, *J* = 8.6 Hz, chloro-benzoyl C2, C6). ^13^C NMR (CDCl_3_) *δ*: 13.27 (1C, **C**H_3_-C-N-), 30.02 (1C, -**C**H_2_-C=O), 55.69 (1C, **C**H_3_-O-), 60.77 (1C, -O-**C**H_2_-CH_2_ONO_2_), 70.19 (1C, -O-CH_2_-**C**H_2_ONO_2_), 101.09 (1C, indole C4), 111.74 (1C, indole C3), 111.80 (1C, indole C6), 114.98 (1C, indole C7), 129.12 (2C, chloro-benzoyl C2, C6), 130.40 (1C, indole C3a), 130.77 (1C, indole C7b), 131.17 (2C, chloro-benzoyl C3, C5), 133.79 (1C, chloro-benzoyl C1), 136.09 (1C, chloro-benzoyl C4), 139.31 (1C, indole C2), 156.08 (1C, indole C5), 168.28 (1C, chloro-benzoyl **C**=O), 170.40 (1C, acetate **C**=O). Anal. Calculated for C_21_H_19_ClN_2_O_7_x0.45H_2_O: C, 55.44; H, 4.41; N, 6.16. Found: C, 55.46; H, 4.54; N, 6.06.

*(+)-2-(Nitrooxy)ethyl 2-(6-methoxynaphthalen-2-yl)propanoate***(4)**: Flash column chromatography (petroleum ether/ethyl acetate, 8/1 and gradually 4/1). White powder, yield, 55%, mp 64–65 °C. IR (nujol) *ν*: 1727 (C=O ester), 1625 (N=O), 1604 (C-C aromatic) cm^−1^. ^1^H NMR (CDCl_3_) *δ*: 1.63 (d, 3H, *J* = 7.1 Hz, C**H_3_**-CH-C=O), 3.95 (s, 3H, -O-C**H_3_**), 4.06–3.87 (m, 1H, CH_3_-C**H**-C=O), 4.48–4.28 (m, 2H, O-CH_2_-C**H_2_**ONO_2_), 4.70–4.54 (m, 2H, O-C**H_2_**-CH_2_ONO_2_), 7.20–7.05 (m, 2H, naphthyl C2, C7), 7.41 (d, 1H, *J* = 6.8 Hz, naphthyl C5), 7.80–7.64 (m, 3H, naphthyl C3, C8, C10). ^13^C NMR (CDCl_3_) *δ*: 18.37 (1C, O=C-CH-**C**H_3_), 45.20 (1C, O=C-**C**H-CH_3_), 55.30 (1C, -O-CH_3_), 60.44 (1C, -O-**C**H_2_-CH_2_ONO_2_), 70.28 (1C, -O-CH_2_-**C**H_2_ONO_2_), 105.56 (1C, naphthyl C5), 119.07 (1C, naphthyl C7), 125.97 (1C, naphthyl C10), 126.01 (1C, naphthyl C9), 127.25 (1C, naphthyl C2), 128.88 (1C, naphthyl C3), 129.26 (1C, naphthyl C8), 133.76 (1C, naphthyl C4), 139.96 (1C, naphthyl C1), 157.71 (1C, naphthyl C6), 174.27 (1C, C=O). Anal. Calculated for C_16_H_17_NO_6_x0.2H_2_O: C, 59.51; H, 5.43; N, 4.34. Found: C, 59.51; H, 5.17; N, 4.65.

*(±)-2-(Nitrooxy)ethyl 2-(3-benzoylphenyl)propanoate***(5)**: Flash column chromatography (petroleum ether/ethyl acetate, 5/1). Viscous transparent liquid, yield 46%. IR (nujol) *ν*: 1740 (C=O ester), 1658, 1636 (N=O), 1598, 1581 (C-C aromatic) cm^−1^. ^1^H NMR (CDCl_3_), *δ*: 1.59 (d, 3H, *J* = 7.1 Hz, O=C-CH-C**H_3_**), 3.86 (q, 1H, *J* = 7.0 Hz, O=C-C**H**-CH_3_), 4.48–4.28 (m, 2H, -O-CH_2_-C**H_2_**ONO_2_), 4.65 (s, 2H, -O-C**H_2_**-CH_2_ONO_2_), 7.86–7.42 (m, 9H, aromatic). ^13^C NMR (CDCl_3_) *δ*: 19.65 (1C, O=C-CH-**C**H_3_), 45.33 (1C, O=C-**C**H-CH_3_), 60.35 (1C, -O-**C**H_2_-CH_2_ONO_2_), 70.64 (1C, -O-CH_2_-**C**H_2_ONO_2_), 128.26 (2C, benzoyl C3, C5), 128, 31 (1C, phenyl C2), 128,73 (2C, benzoyl C2, C6), 129, 35 (1C, phenyl C4), 130, 84 (1C, phenyl C5), 131, 22 (1C, phenyl C6), 131,74 (1C, benzoyl C4), 137,12 (1C, benzoyl C1), 140.78 (1C, phenyl C1), 142.61 (1C, phenyl C3), 175.12 (1C, O-**C**=O), 190,83 (1C, **C**=O ketone). Anal. Calculated for C_18_H_17_NO_6_x0.2H_2_O: C, 62.32; H, 5.05; N, 4.03. Found: C, 62.32; H, 5.13; N, 4.19.

*(E)-2-(Nitrooxy)ethyl cinnamate***(6**): Flash column chromatography (petroleum ether/ethyl acetate, 40/1). Viscous transparent liquid, yield 42%. IR (film) *ν*: 1717 (C=O ester), 1636 (N=O), 1578, 1496 (C-C aromatic) cm^−1^. ^1^H NMR (CDCl_3_), *δ*: 4.68–4.46 (m, 2H, -O-CH_2_-C**H_2_**ONO_2_), 4.98–4.70 (m, 2H, -O-C**H_2_**-CH_2_ONO_2_), 6.47 (d, 1H, 16.0 Hz, -C**H**-CH-C=O), 7.53–7.40 (m, 3H, aromatic C3, C4, C5), 7.68–7.54 (m, 2H, aromatic C2, C6), 7.75 (d, 1H, 16.0 Hz, -CH-C**H**-C=O). ^13^C NMR (CDCl_3_) *δ*: 59.78 (1C, O-**C**H_2_-CH_2_ONO_2_), 70.44 (1C, O-CH_2_-**C**H_2_ONO_2_), 116.24 (1C, -CH-**C**H-C=O), 126.05 (1C, phenyl C1), 125.87 (2C, phenyl C2, C6), 126.33 (2C, phenyl C3, C5), 128.46 (1C, phenyl C4), 146.50 (1C, -**C**H-CH-C=O), 167.75 (1C, -CH-CH-**C**=O). Anal Calculated for C_11_H_11_NO_5_: C, 55.70; H, 4.67; N, 5.90. Found: C, 55.28; H, 4.37; N, 5.95.

*(E)-2-(Nitrooxy)ethyl 3-(3,5-di-tert-butyl-4-hydroxyphenyl)acrylate***(7)**: Flash column chromatography (petroleum ether/ethyl acetate, 20/1). White powder, yield 71%, mp 80 °C. IR (nujol) *ν*: 3569 (O-H), 1711 (C=O ester), 1630 (N=O), 1596 (C-C aromatic) cm^−1^. ^1^H NMR (CDCl_3_), *δ* (ppm): 1.48 (s, 18H, -CH_3_), 4.55–4.47 (m, 2H, O-CH_2_-C**H_2_**ONO_2_), 4.80–4.72 (m, 2H, O-C**H_2_**-CH_2_ONO_2_), 5.59 (s, 1H, Ar-OH), 6.32 (d, 1H, *J* = 15.9 Hz, -CH=C**H**-C=O), 7.40 (s, 2H, phenyl), 7.66 (d, 1H, *J* = 15.9 Hz, -C**H**-CH-C=O). ^13^C NMR (CDCl_3_) *δ*: 30.12 (6C, C-(**C**H_3_)_3_), 34.32 (2C, **C**-(CH_3_)_3_), 59.96 (1C, O-**C**H_2_-CH_2_ONO_2_), 70.76 (1C, O-CH_2_-**C**H_2_ONO_2_), 113.29 (1C, -CH-**C**H-C=O), 125.47 (1C, phenyl C1), 125.65 (2C, phenyl C2, C6), 136.40 (2C, phenyl C3, C5), 147.19 (1C, -**C**H-CH-C=O), 156.44 (1C, phenyl C4), 166.92 (1C, -CH-CH-**C**=O). Anal. Calculated for C_19_H_27_NO_7_: C, 62.45; H, 7.45; N, 3.83. Found: C, 62.03; H, 7.17; N, 4.18.

*(E)-4-Nitrophenethyl 3-(3,5-di-tert-butyl-4-hydroxyphenyl)acrylate***(8):** Flash column chromatography (petroleum ether/ethyl acetate, 10/1). Yellow powder, yield 60%, mp 156–157 °C. IR (nujol) *ν*: 3626 (O-H), 1706 (C=O ester), 1629 (N=O), 1606, 1591 (C-C aromatic) cm^−1^. ^1^H NMR (CDCl_3_) *δ*: 1.49 (s, 18H, -CH_3_), 3.16 (t, 2H, O-CH_2_-C**H_2_**-Ar), 4.50 (t, 2H, O-C**H_2_**-CH_2_-Ar), 5.54 (s, 1H, Ar-OH), 6.27 (d, 1H, *J* = 15.9 Hz, -CH=C**H**-C=O), 7.38 (s, 2H, phenyl C2, C6), 7.46 (d, 2H, *J* = 7.5 Hz, nitrophenyl C2, C6), 7.66 (d, 1H, *J* = 15.9 Hz, -C**H**=CH-C=O), 8.21 (d, 2H, *J* = 7.5 Hz, nitrophenyl C3, C5). ^13^C NMR (CDCl_3_) *δ*: 30.12 (6C, C-(**C**H_3_)_3_), 34.31 (2C, **C**-(CH_3_)_3_), 35.13 (1C, O-CH_2_-**C**H_2_-Ar), 63.64 (1C, O-**C**H_2_-CH_2_-Ar), 113.89 (1C, -CH-**C**H-C=O), 123.60 (2C, phenyl C2, C6), 125.51 (2C, nitrophenyl C3, C5), 125.53 (1C, phenyl C1), 129.76 (2C, nitrophenyl C2, C6), 136.40 (2C, phenyl C3, C5), 145.89 (1C, -**C**H-CH-C=O), 146.52 (1C, nitrophenyl C4), 146.87 (1C, nitrophenyl C1), 156.32 (1C, phenyl C4), 167.20 (1C, -CH-CH-**C**=O). Anal. Calculated for C_25_H_31_NO_5_: C, 70.57; H, 7.34; N, 3.29. Found: C, 70.34; H, 7.67; N, 3.51.

*(±)-2-(Nitrooxy)ethyl 6-hydroxy-2,5,7,8-tetramethylchroman-2-carboxylate***(9):** Flash column chromatography (petroleum ether/ethyl acetate, 40/1). White powder, yield 54%, mp 95–97 °C. IR (nujol) *ν*: 3532 (O-H), 1741 (C=O ester), 1649, 1634 (N=O) cm^−1^. ^1^H NMR (CDCl_3_), *δ* (ppm):1.65 (s, 3H, chromane C1), 2.00–1.86 (m, 1H, chromane C2 axial), 2.20, 2.19, 2.09 (3s, 9H, -CH_3_ aromatic), 2.52–2.41 (m, 1H, chromane C2 equatorial), 2.74–2.53 (m, 2H, chromane C3), 4.44–4.24 (m, 2H, -O-CH_2_-C**H_2_**ONO_2_), 4.60–4.47 (m, 2H, -O-C**H_2_**-CH_2_ONO_2_). ^13^C NMR (CDCl_3_) *δ*: 11.17, 11.71, 12.13 (3C, -**C**H_3_ aromatic), 20.87 (1C, chromane C3), 25.32 (1C, chromane C1-**C**H_3_), 30.61 (1C, chromane C2), 60.46 (1C, Ο-**C**H_2_-CH_2_ΟNΟ_2_), 70.28 (1C, Ο-CH_2_-**C**H_2_ΟNΟ_2_), 77.19 (1C, chromane C1), 116.80 (1C, chromane C4), 118.42 (1C, chromane C7), 121.24 (1C, chromane C8), 122.64 (1C, chromane C5), 145.40 (1C, chromane C6), 145.47 (1C, chromane C9), 173.63 (1C, C=O). Anal. Calculated for C_16_H_21_NO_7_ x2H_2_O: C, 51.20; H, 6.71; N, 3.73. Found: C, 51.31; H, 6.47; N, 3.87.

### 3.3. Biological Evaluation

κ-Carrageenan and lipoxygenase type I-B from soybean were purchased from Sigma (St. Louis, MO, U.S.A.). For the in vivo experiments, Wistar rats (180–220 g, 3–4 months old) were kept in the Centre of the School of Veterinary Medicine (EL54 BIO42), Aristotelian University of Thessaloniki, which is registered by the official state veterinary authorities (presidential degree 56/2013, in harmonization with the European Directive 2010/63/EEC). The experimental protocols were approved by the Animal Ethics Committee of the Prefecture of Central Macedonia (no. 270079/2500).

#### 3.3.1. Effect on Plasma Cholesterol, Triglyceride and LDL-Cholesterol Levels

Hyperlipidaemia was induced by the *i.p.* administration of Triton WR 1339 (200 mg/kg) to rats. The examined compounds (0.15 mmol/kg) were administered *i.p.* one hour later. Blood was taken from the aorta after 24 h, for the determination of plasma total cholesterol, LDL-cholesterol and triglyceride concentrations, using commercial kits [13].

#### 3.3.2. In Vitro NO Release

Due to their poor aqueous solubility, compounds **1**, **3-7** and **9** were dissolved in 7/3 (*v*/*v*) mixture of DMSO/water at various concentrations and incubated overnight at room temperature in the presence of cadmium. *S*-nitroso-*N*-acetylpenicillamine (SNAP) was dissolved either in DMSO/water 7/3 or in water and treated as above.

Aliquots were taken from each sample and added to an equal volume of *N*-naphthylaminoethylamine (0.2%) and sulfanilamide (2%) solution in 3 N hydrochloric acid (Griess reagent). Appropriate sodium nitrite solutions in the same DMSO/water mixture were used for the construction of the standard curve and values were identical to those obtained with aqueous solutions. Nitric oxide release was estimated spectrophotometrically (540 nm) [9]. NO release from SNAP (100 μM in DMSO/water 7/3 and in water) was found to be 56.3 and 56.7 μΜ, respectively, further verifying that the addition of DMSO did not influence the experiment.

#### 3.3.3. In Vitro Evaluation of Lipoxygenase Activity

The reaction mixture contained the examined compounds (in absolute ethanol, 1–300 μM), soybean lipoxygenase (in saline, 250 u/mL) and sodium linoleate (100 μM), in Tris–HCl buffer, pH 9.0. The reaction was monitored for 7 min at 28 °C, recording the absorbance at 234 nm. Nordihydroguaiaretic acid (NDGA) was used as a reference.

For the estimation of the type of inhibition, the described experiments were repeated, using sodium linoleate at a concentration (1 mM) higher than the saturating substrate concentration [23].

#### 3.3.4. In Vitro Lipid Peroxidation

The peroxidation of heat-inactivated (90°, 90 s) rat liver microsomal fraction was induced by ascorbic acid (0.2 mM) and ferrous sulphate (10 μΜ). The studied compounds, in dimethylsulfoxide, were added at concentrations 1 μM–1 mM. Aliquots were taken from the incubation mixture (37 °C) for 45 min. Lipid peroxidation was assessed spectrophotometrically (535/600 nm) as 2-thiobarbituric acid reactive material. All compounds and solvents were found not to interfere with the assay [25].

#### 3.3.5. In Vitro Interaction with the Stable Radical 1,1-Diphenyl-2-Picrylhydrazyl (DPPH)

Compounds (in absolute ethanol, final concentrations 25–200 μM) were added to an ethanolic solution of DPPH (final concentration 200 μM) at room temperature (22 ± 2 °C). Absorbance (517 nm) was recorded after 30 min.

#### 3.3.6. Carrageenan-Induced Paw Oedema

The tested compounds (in water with a few drops of Tween 80) were given *i.p.* (0.15 mmol/kg) to rats, just after the *i.d.* injection of 0.1 mL of an aqueous carrageenan solution (1% *w*/*v*) in the hind paw of rats. The produced oedema, after 3.5 h, was estimated as paw weight increase [24].

## 4. Conclusions

There are a number of reports in the literature concerning derivatives of NSAIDs with NO releasing ability. Those compounds were designed mainly as gastroprotective agents, counteracting the gastric irritation caused by the classical NSAIDs [26]. In addition, various derivatives of antioxidants, such as butylated hydroxytoluene, vitamin E and trolox, acting as NO donors, have been synthesised and tested as vasodilating agents in in vitro experiments [27,28,29].

In the present investigation, the designed new compounds were synthesised and tested for hypolipidaemic activity in vivo, a process that is close to the medical practice. This activity is also correlated with their in vivo effect on acute inflammation, their ability to act as NO donors, lipoxygenase inhibitors and antioxidants in vitro. Among the tested compounds, the naproxen derivative **4** demonstrated strong hypolipidaemic (80% reduction of total cholesterol) and anti-inflammatory (70% reduction of rat paw oedema) activity. The butylated hydroxycinnamic acid analogue **7** is a potent hypolipidaemic (66% reduction of total cholesterol), anti-inflammatory (55% reduction of rat paw oedema) and antioxidant (lipid peroxidation inhibition, IC_50_ 41 μΜ) compound. The ester of butylated hydroxycinnamic acid with 4-nitrophenylethyl alcohol **8** inhibits lipoxygenase (IC_50_ 10.5 μΜ) and is an anti-inflammatory agent (51% reduction of rat paw oedema).

The successful combination of antioxidant, anti-inflammatory and NO releasing activities is important for compounds that would acquire a series of biological properties able to prevent or restore a number of pathological changes in conditions like cardiovascular and inflammatory disorders. We hope that the described multifunctional compounds may assist towards the development of agents against multicausal disorders, which otherwise would require multiple drug therapies to address the various pathological disturbances.

## Figures and Tables

**Table 1:** Effect of the nitrooxy compounds, simvastatin, ibuprofen and ketoprofen on Triton WR1339 (tyloxapol) induced hyperlipidaemia.

Compound	Dose (*i.p.*) (μmol/kg)	% Reduction		
		TC ^a^	TG ^b^	LDL-C ^c^
**1**	150	62.2 **	30.1 **	80.0 *
**3**	150	71.7 **	31.8 **	62.3 *
**4**	150	78.2 ***	38.9 ***	84.6 ***
**5**	150	46.6 **	40.2 ***	57.8 ***
**6**	150	70.9 ***	54.4 ***	74.8 ***
**7**	150	65.5 **	45.7 ***	60.6 *
**9**	150	55.4 ***	30.0 ***	65.0 ***
**Simvastatin**	150	73.0 ***	-	70.0 ***
**Ibuprofen**	300	41.0 ***	38.0 ***	41.6 ***
**Naproxen**	500	53.0 ***	43.5 ***	25.5 ***

^a^ TC: Total cholesterol; ^b^ TG: Triglycerides; ^c^ LDL-C: LDL cholesterol. Tyloxapol: 200 mg/kg, *i.p.* Significant difference from hyperlipidaemic control group: * *p* < 0.01, ** *p* < 0.005, *** *p* < 0.001 (Student’s *t* test).

**Table 2:** In vitro nitrogen monoxide release.

Compound	ΝΟ Release (μΜ)/Compound						
	1	3	4	5	6	7	9
500 μM	116	102	73	100	98	77	93
250 μM	82	58	37	57	51	38	49
125 μM	40	28	17	26	22	17	23
62.5 μM	17	13	7	11	11	6.5	10
31.25 μM	7	5	2.5	4	5	2	3

All determinations (using the Griess reagent) were performed at least in triplicate and the standard deviation was always within ±10% of the mean value.

**Table 3:** Effect of compounds **1**–**9,** ibuprofen, ketoprofen, trolox and NDGA on lipoxygenase ^a^, and their lipophilicity (clog*P*) ^b^.

Compound	IC_50_ (μM)	clog*P*
**1**	46	4.65
**2**	24	5.92
**3**	107	5.15
**4**	>>300	3.78
**5**	86	3.73
**6**	220	3.18
**7**	44	6.16
**8**	10.5	7.44
**9**	120	4.16
**Ibuprofen**	200	
**Ketoprofen**	220	
**Trolox**	>>300	3.10
**NDGA**	1.3	

^a^ After 7 min of incubation; NDGA: nordihydroguaiaretic acid. ^b^ clog*P*: Clog*P* for windows v. 4.0, BioByte Corp (BioByte Corporation, Claremont, CA, U.S.A.).

**Table 4:** Interaction of compounds **7**–**9** and trolox, at various concentrations, with DPPH (200 μΜ) ^a^ and their effect on lipid peroxidation ^b^.

Compound	Percent Interaction with DPPH			Inhibition of Lipid Peroxidation IC_50_ (μΜ)
	200 μΜ	100 μΜ	50 μΜ	
**7**	87	49	21	41
**8**	88	57	23	150
**9**	92	90	55	2.3
**Trolox**	92	90	38	25

^a^ After 30 min of incubation. ^b^ After 45 min of incubation. Trolox: 6-hydroxy-2,5,7,8-tetramethylchroman-2-carboxylic acid. All determinations were performed at least in triplicate and the standard deviation was always within ±10% of the mean value.

**Table 5:** Effect of compounds **1**–**9**, ibuprofen, indomethacin, ketoprofen and naproxen on carrageenan-induced rat paw oedema ^a^.

Compound	% Oedema Reduction
**1**	76 **
**2**	53 *
**3**	29 **
**4**	70 **
**5**	75 **
**6**	61 **
**7**	55 **
**8**	51 **
**9**	57 **
**Ibuprofen**	36 *
**Indomethacin**	42 *
**Naproxen**	11 *
**Ketoprofen**	47 *

^a^ The effect on oedema is expressed as percent oedema reduction in comparison to controls, which received carrageenan only. All compounds were administered *i.p*. at 0.15 mmol/kg of body weight. Significant difference from control: * *p* < 0.005, ** *p* < 0.001 (Student’s *t* test).
